# Association between mobile work and work ability: a longitudinal study under the impact of the COVID-19 pandemic

**DOI:** 10.1007/s00420-022-01849-5

**Published:** 2022-03-17

**Authors:** Ines Berling, Marlies Jöllenbeck, Tjorven Stamer, Elke Ochsmann

**Affiliations:** Luebeck Institute of Occupational Health (LIOH), University of Lübeck, Ratzeburger Allee 160, 23562 Lübeck, Germany

**Keywords:** Mobile work, Work ability, Occupational health, Remote work

## Abstract

**Objective:**

This study examines the effect of mobile work on work ability as direct predictor and as factor moderating workplace stressors and resources. Originally, the study focused on the effects of mobile work on work ability in a mobile test group compared to office workers. As the study period of 1 year collided with the beginning of the COVID-19 pandemic and lockdown restrictions, we can now explore the association of mobile work and work ability before and during the first year of the COVID-19 pandemic.

**Methods:**

This longitudinal, exploratory study took place in a medium-sized company in the social insurance sector in Germany. We used a mixed-methods design (online survey and focus group interviews) with two survey dates 1 year apart (T0: summer/autumn 2019 (before COVID-19 pandemic), T1: summer 2020 (during COVID-19 pandemic, after first strict lockdown)). Quantitative data, which are reported here, were collected by means of an online questionnaire, which includes questions on mobile work and validated measures for work-related stressors and resources and work ability. Non-parametric tests, regression analysis, and logistic regression models were used for data analysis.

**Results:**

The linked data set of both survey dates includes *N* = 102 persons (men: 37%, mean age: 41–50 years). Interestingly, we found an improvement in work ability over the course of the study (*p* = 0.007), although it included the first and most drastic COVID-19 restrictions in Germany. Before the pandemic, correlations between work ability and work-related stressors (e.g., work–privacy conflicts) and resources (e.g., sense of community) were evident. Some of these factors are moderated by mobile work. During the COVID-19 pandemic, mobile work was identified as independent factor for work ability. In addition, technology competence conviction gained importance as a personal resource in our cohort.

**Conclusions:**

Work ability can be influenced by many factors. Our study, which allowed for a comparison of work ability before and during COVID-19 pandemic, suggests that mobile work can be especially helpful to maintain work ability in times of change. Our findings support the notion that—under normal conditions—mobile work can influence work ability via work-related stressors and resources. In times of changes, it can have an independent effect on work ability. It must be assumed that the effects can be highly individual or context-specific.

## Introduction

In the face of aging workforces, VUCA work environments (volatility, uncertainty, complexity, and ambiguity), and last but not least, a global pandemic, the work ability of employees is a valuable good for economies and societies. Therefore, it seems important to focus research on factors associated with work ability.

Having good work ability means that there is a balance between the individual’s mental and physical capacity and the work requirements set by the company. In the best case, employees can meet the requirements with a reasonable amount of effort and in good quality (Ilmarinen 2009). But apart from that, work ability is an entity which can be influenced by multiple factors. Ilmarinen (2009) described some of these factors in his house of work ability, where four floors represent four aspects that are important for developing and maintaining work ability. These include mental and physical health, professional and social competence, values, and working conditions. Especially the latter factors have been under scrutiny in the context of occupational or work-related measures to improve work ability. A study by Weber et al. (2021), for example, found that improvements in workplace factors can have a beneficial effect on work ability. Another study of Burr et al. (2022) found only small effects of workplace factors for the work ability of the general workforce, but reports that the relevance of these factors can increase in workforce-subgroups. Therefore, it can be assumed that a regular assessment and eventually improvement of workplace factors or working conditions can increase the odds for a better work ability of the respective workforce.

In this context, we focused our research on the association between mobile work and work ability. With regard to mobile work, economies, societies, companies, and employees experienced meaningful changes over the last decade, which currently culminated in contact restrictions because of COVID-19 pandemic, which also affected workplaces and led to an abrupt rise in the use of mobile technologies at German workplaces. In Germany, most companies followed a strategy of working on-site before COVID-19 pandemic, so many people began working from home (Demmelhuber et al. 2020) only with the beginning of the first COVID-19-related lockdown. This development now seems to gain ground.

Despite its increasing relevance, the term “mobile work” is currently not clearly defined in the literature and is used interchangeably (Harker Martin and MacDonnell 2012; Mojtahedzadeh et al. 2021). In most cases, though, mobile work is characterized by the fact that information and communication technologies (ICT) are used from outside the workplace or office to get the work done, establish a connection to the office, to other colleagues, or to customers. Mobile work is, therefore, usually not tied to a fixed location and a fixed working time and can theoretically be performed almost anywhere and at any time (Bailey and Kurland 2002; Deutscher Bundestag 2017; Gajendran and Harrison 2007). In this study, the term mobile work is also defined broadly and includes all work performed outside the office: remote work, field service, working in co-working spaces and public places, working during business travels, working during office hours and beyond. Of course, during COVID-19 lockdown, the possible applications of mobile work mainly focused on remote work from home.

Mobile work can have an impact on work ability by altering societal or personal circumstances or stressors or by addressing personal motives. Persons can have different motives for mobile work (e.g., avoidance of commuting, tending to non-work demands, and personal illness) (Thompson et al. 2021). If mobile work addresses these motives, it increases work ability. In addition, work performance, commitment and employee loyalty can be positively influenced by mobile work (Harker Martin and MacDonnell 2012; Waltersbacher et al. 2019). Note though that these benefits are highly individual, which is probably one of the reasons that many surveys report positive as well as negative effects of mobile work on individuals (often in one person) (Mazmanian et al. 2013).

Apart from fulfilling personal or societal motives or needs, mobile work can affect work ability by changing relevant workplace stressors and resources (e.g., work–life conflict, quality of leadership, social cohesion, and quantitative workload) and thusly have an indirect impact on work ability. Positive effects of mobile work before the pandemic, e.g., were associated with a quieter working atmosphere, more self-determination, a better work–life balance and an increased autonomy (Fonner and Roloff 2010; Gisin et al. 2013; Mann and Holdsworth 2003; Waltersbacher et al. 2019). While these effects, too, are probably highly individual, a meta-analysis by Gajendran and Harrison (2007), found small but overall positive associations between mobile work and workplace factors such as perceived autonomy or lower work–life conflict.

Hence, mobile work can influence work ability by addressing individual and/or societal circumstances, or by altering work-related stressors and resources. Both aspects could be used to create healthy workplaces. Our study tries to highlight possible changes in the interaction between mobile work and work ability under “normal” conditions and in the context of the COVID-19 pandemic. Mobile work was examined as independent variable for work ability and also as variable related to work-related stressors and resources. As theoretical model, the job demands resources model (Demerouti et al. 2001) has been used as underlying model to examine the effect of work-related stressors and resources on work ability before (e.g., Viotti et al. 2017), and was also used in this study.

Workplace resources like social support are beneficial for coping with workplace-related stress. They are usually positively correlated with lower levels of all mobile working challenges, such as work–home interference, ineffective communication, procrastination and loneliness (Wang et al. 2021). They can also have a positive impact on health-associated outcomes (Oakman et al. 2020). A study from Latin America examined the relationship between mobile work, work-related stress, and work–life balance and concluded that working from home during times of pandemic was associated with greater perceptions of stress, poorer work–life balance and job satisfaction, and increased productivity (Sandoval-Reyes et al. 2021). In contrast, an Austrian study identified that people who work from home report lower perceived productivity. However, working from home led to an increased quality of life (Weitzer et al. 2021).

With these aspects in mind, the relevance of research on mobile work and work ability should be obvious. Results can be used to design a “healthy” environment and beneficial working conditions for mobile work. This was one of the aims of this study which started in 2019 as an evaluation of a test group of mobile workers vs. office workers in a company and commenced in 2020 under the influence of the COVID-19 pandemic. Although the survey generally took place in the context of mobile work, the extent to which most participants had to use mobile work because of the COVID-19 pandemic was an unexpected development. We considered this by adjusting our analysis for potential (pandemic-related) confounders.

### Research questions

**Fig. 1:**
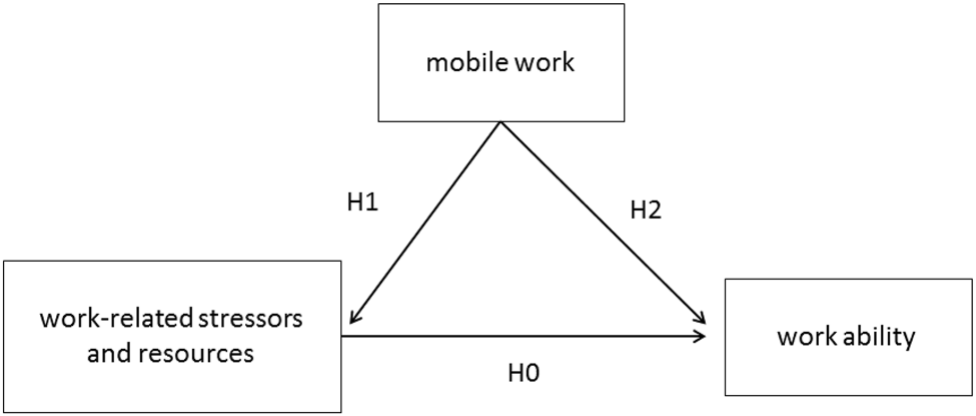
Hypothetical associations between mobile work, work-related stressors and resources, and work ability. H0: work-related stressors and resources are associated with work ability. H1: mobile work can be associated with work-related stressors and resources. H2: mobile work can be directly associated with work ability

In this explorative approach, we examine the association of work-related stressors and resources on work ability at T0 and T1 (H0). Second, we assume that mobile work can affect work ability by addressing and/or changing work-related stressors and resources. We examined our cohort in the light of this hypothesis (H1). Finally, we examined if mobile work can have a direct effect on work ability, by addressing personal and societal needs and motives (H2) (Fig. 1).

## Methods

### Study design

To explore the presented research topic, a longitudinal, exploratory study was conducted in a mixed-methods design. There were two survey time points for the quantitative and qualitative part, which took place in the time frame from 2019 and 2020. This report focuses on the quantitative results (T0 quantitatively: July/August 2019; T1 quantitatively: July/August 2020), which were gathered by an online survey. The beginning of the COVID-19 pandemic falls within the survey period, so consequently the measurements took place before (T0) and during (T1) the COVID-19 pandemic.

### Setting and participants

The survey took place in a medium-sized company in the social insurance sector, which initially wanted to test and establish mobile work due to structural alteration measures. The company has four locations in northern Germany and employs 287 people. The employees are divided into the areas of administration/internal service, consulting/supervision of companies and performance field clerk. Their work mainly involves paperwork with cognitive content. The gender and age distribution throughout the company is as follows: 38% men, 62% women, 0% divers, average age 47.7 years (standard deviation: 12.0 years).

### Participant recruitment and ethical principals

Employees were informed about the quantitative part of the study by mail and personally by their supervisors and were asked to participate. The recruitment of participants for the focus group interviews was organized internally by the company. Participation in the study was voluntary. In the course of the two online surveys, participants gave themselves pseudonyms, so that they could be identified for longitudinal analyses. There was no incentive system for participation and no compensation for the time expenditure. However, both parts of the study were allowed to be conducted during regular working hours.

A positive ethical vote of the University of Lübeck is available. The study was conducted according to scientific standards in accordance with the Guidelines for Assuring Good Scientific Practice (Deutsch Forschungsgemeinschaft 2019).

The participants of the study were comprehensively informed about the aim and purpose of the study. This was done both orally and in writing. Data protection was fully considered.

### Quantitative survey

The online questionnaire was created using the scientific platform SoSci Survey (Leiner 2019) and a link was sent to the participants via e-mail. The link to the questionnaire was active for 1 month at both measurement times T0 and T1. The questionnaire contains various validated measurement instruments, of which only the Work Ability Index (WAI), the Copenhagen Psychosocial Questionnaire (COPSOQ), the General Self-Efficacy Scale and the GESIS short scale for measuring technology commitment were used to answer the research question of this report. In addition, sociodemographic variables (age, sex, working area, working time, leadership, and children in the household) and the usage of mobile work were assessed.

### Work ability index (WAI)

The work ability index is a validated measurement instrument for assessing self-reported work ability (Hasselhorn and Freude 2007). It was developed in the early 1980s by the Finnish Institute of Occupational Medicine. The question about current work ability in comparison to the best work ability ever achieved was used (0 = completely unable to work, 10 = currently the best work ability).

### Copenhagen psychosocial questionnaire (COPSOQ)

The COPSOQ is a measurement instrument for the assessment of mental stress and strain at the workplace. It relates to the job demand resources model (Demerouti et al. 2001). For this research project, the short German standard version of the COPSOQ from 2019 was used (Nübling et al. 2019). A 5-point Likert scale with the response options “always”, “often”, “sometimes”, “rarely”, and “never/almost never” or “to a very high degree”, “to a high degree”, “to some extent”, “to a low degree”, and “to a very low degree” is available as a response option. The scaling 1 (always/to a high degree) to 5 (never/to a very low degree) was recoded into values from 0 to 100, whereby for the coding direction it was necessary to consider whether the respective item is a resource or a stressor. In general, a high stressor and/or a low resource were associated with a numerical high score. The items can be assigned to superordinate scales. The following scales were assessed: quantitative demands, emotional demands, work–privacy conflicts, trust and justice, dissolution, role conflicts, influence at work, meaning of work, predictability of work, role clarity, quality of leadership, support at work, amount of social contacts and sense of community. The Cronbach’s alpha of the scales ranges from 0.686 to 0.977.

### General self-efficacy scale

The measurement instrument for general self-efficacy expectations by Schwarzer and Jerusalem, (1995) consists of ten validated items with a 4-point Likert scale. An exemplary item is: “I can remain calm when facing difficulties because I can rely on my coping abilities.” The scale ranges from 1 (not true) to 4 (true exactly). For evaluation, the answers of all ten items are summed up, resulting in a score between 10 and 40. A high score represents a good self-efficacy. Cronbach’s alpha is 0.76–0.90. The measuring instrument records the extent to which a person has confidence in his/her own competence to master a difficult situation.

### GESIS short scale for measuring technology commitment

The short scale for assessing technology readiness by Neyer et al. (2016) is a validated measurement instrument for examining personal attitudes and the use of modern technology. Four items regarding technology competence conviction were used and answered on a 5-point Likert scale. The scale ranges from 1 (not at all true) to 5 (completely true). An exemplary item is: “When dealing with modern technology, I am often afraid of failing.” For evaluation, the values of the items can be summed up, resulting in a score between 4 and 20. A high value means a low technology competence conviction. Cronbach’s alpha of the questionnaire ranges from 0.74 to 0.84 (Neyer et al. 2016).

### Definition of mobile work in the context of this survey

Mobile workers in this study include persons who work from home for at least 1 day/week and/or work in field service for at least 1 day/week and/or have business travel activities for at least 1 day/week.

### Statistical analysis

The quantitative data were analyzed using the statistical program SPSS 25. The data from both measurement points were linked using a pseudonymization code. In addition, a plausibility check was carried out based on the variables “age”, “gender”, “working area”, “full-time/part-time”, and “leader/no leader”. The Kolmogorov–Smirnov test was used to test for normal distribution of the data. Since the measured values are not normally distributed, Mann–Whitney-*U*-tests were used for group comparisons and Wilcoxon-tests for comparison of two connected samples. Furthermore, Spearman correlations were conducted to check for significant relationships between stressors/resources and self-reported work ability. An alpha error level of *p* < 0.05 was used for all statistical tests.

The Spearman correlation results associated with a statistical *p* value < 0.05 were used to create models for logistic regression analysis for time points T0 and T1. As outcome variable, the dependent variable “work ability” was dichotomized with a cutoff value of 7.0 (El Fassi et al. 2013). The independent variables of the logistic regression models consisted of the interesting variable (mobile work), confounders (age, sex, working area, leadership, child/children under the age of 12 in the household) and workplace-related stressors and resources (e.g., work–life conflict). Only stressor and resource variables which fulfilled the statistical criterion in the correlation analyses (see above) were included in the logistic regression models. All independent variables were dichotomized using an approximate half distribution. To reduce model size, backwards selection (likelihood ratio) was used for logistic regression analyses. The selection process was restricted to 20 iterations. Again, *p* values < 0.05 were defined as inclusion criteria, *p* values < 0.10 were defined as exclusion criteria. Results of the two logistic regression analyses were reported as Odds Ratio (OR) with corresponding 95% confidence intervals (95% CI).

## Results

Study participants: of 287 persons in the company, *N* = 183 persons participated in the quantitative survey at T0 and *N* = 144 persons at T1. In comparison with other employee surveys, this response rate was satisfactory. Merging the data sets resulted in *N* = 102 connected cases between T0 and T1. At T0 and T1, most of the participants were between 51 and 60 years old. Thirty-seven percent of the participants were male. For further sociodemographic information, see Table 1. In general, the composition of the sample is, regarding age and sex, very similar to the human resources (HR) data of the surveyed company and was, therefore, regarded as representative.

**Table 1:** Sociodemographic information on the linked sample at T0 and T1

	T0 (*N* = 102) (%)	T1 (*N* = 102) (%)	Company (*N* = 287) (%)
*Age*			
18–20 years	0.0	0.0	2.4
21–30 years	5.9	4.9	8.7
31–40 years	17.6	15.7	17.4
41–50 years	27.5	25.5	22.6
51–60 years	45.1	49.0	36.9
61 years and older	3.9	4.9	11.8
*Sex*			
Male	36.6	37.3	38.0
Female	63.4	62.7	62.0
*Working area*			
Administration/internal service	58.3	60.4	–
Consulting and supervision of companies	37.5	32.3	–
Performance field clerk	4.2	7.3	–
*Full-time*			
Yes	67.6	68.3	–
No	32.4	31.7	–
*Leadership*			
Yes	20.6	25.7	–
No	79.4	74.3	–
*Mobile work*			
Yes	57.8	88.2	–
No	42.2	11.8	–

Table 2 presents the sociodemographic data of mobile and non-mobile workers for both time points. Most mobile workers are between 51 and 60 years old, work full-time and are not in management positions. The non-mobile workers were mostly female and all work in administration. For more information, see Table 2. Sociodemographic and work-related factors were unevenly distributed and could, therefore, cause bias for the results and interpretation of data. To avoid this, they were included as confounders in final regression models.

**Table 2:** Sociodemographic information on mobile workers and non-mobile workers, T0 and T1

	T0 (%)		T1 (%)	
	Mobile work (*N* = 59)	No mobile work (*N* = 43)	Mobile work (*N* = 90)	No mobile work (*N* = 12)
*Age*				
18–20 years	0.0	0.0	0.0	0.0
21–30 years	8.5	2.3	4.4	8.3
31–40 years	15.3	20.9	15.6	16.7
41–50 years	23.7	32.6	24.4	33.3
51–60 years	49.2	39.5	50.0	41.7
61 years and older	3.4	4.7	5.6	0.0
*Sex*				
Male	51.7	16.3	40.0	16.7
Female	48.3	83.7	60.0	83.3
*Working area*				
Administration/internal service	25.9	100.	54.8	100.0
Consulting and supervision of companies	66.7	0.0	36.9	0.0
Performance field clerk	7.4	0.0	8.3	0.0
*Full-time*				
Yes	78.0	53.5	68.9	63.6
No	22.0	46.5	31.1	36.4
*Leadership*				
Yes	30.5	7.0	29.2	0.0
No	69.5	93.0	70.8	100.0

Description of mobile work: understandably, in the course of the COVID-19 pandemic, the frequency and duration of mobile work changed between T0 and T1. At T0, mobile workers worked from home for 1 day/week. At T1, they worked for 4 days/week from home (Table 3).

**Table 3:** Frequencies of remote work, field service, and business travel, T0 and T1

*T0 (N = 102)*						
Days per week	0	1	2	3	4	5 and more
Remote work (days/week)	52.5%	18.6%	8.5%	6.8%	8.5%	5.1%
Field service (days/week)	15.3%	25.4%	39.0%	11.9%	8.5%	0.0%
Business travel (days/month)	32.2%	22.0%	15.3%	11.9%	8.5%	10.2%
*T1 (N = 102)*						
Days per week	0	1	2	3	4	5 and more
Remote work (days/week)	3.3%	3.3%	11.1%	18.9%	34.4%	28.9%
Field service (days/week)	38.6%	23.9%	26.1%	11.4%	0.0%	0.0%
Business travel (days/month)	49.4%	15.7%	14.6%	5.6%	9.0%	5.6%

While approximately half of the participants (57.8%) were mobile workers at T0, a much higher percentage than initially expected (88.2%) worked mobile at T1 due to the COVID-19 pandemic. The non-mobile participants continued to work in the office because their work was not feasible to perform from home or they did not want to work mobile. Note though that work was organized for all employees to include at least 1 day/week at the office, even during COVID-19 pandemic.

Workplace stressors/resources: Tables 4 and 5 describe the mean values of work-related stressors and resources for the whole sample, and for mobile workers compared to non-mobile workers. All in all, work-related stressors decreased between T0 and T1 (with the exception of dissolution/blurred boundaries), while resources became better or remained stable (e.g., meaning of work, quality of leadership, Table 4).

**Table 4:** Stressors and resources for the linked sample, T0 and T1 (Wilcoxon; paired sample)

	*n*	T0	T1	*p*
		Mean (SD)	Mean (SD)	
*Stressors*				
Quantitative demands	102	46.1 (20.5)	41.7 (18.1)	0.003*
Emotional demands	102	50.7 (27.1)	48.4 (26.2)	0.248
Work–privacy conflicts	99	23.4 (21.9)	16.5 (18.8)	< 0.001*
Trust and justice	96	45.3 (18.2)	39.7 (17.9)	< 0.001*
Dissolution	100	16.3 (18.6)	17.8 (19.6)	0.532
Role conflicts	100	36.1 (20.9)	32.9 (19.6)	0.124
*Resources*				
Influence at work	98	51.2 (22.3)	47.0 (19.4)	.041*
Meaning of work	100	46.8 (37.9)	23.0 (16.4)	< .001*
Predictability of work	101	46.8 (21.2)	38.1 (17.9)	< .001*
Role clarity	100	29.4 (21.4)	23.9 (18.1)	0.002*
Quality of leadership	99	52.1 (27.2)	41.6 (20.4)	< .001*
Support at work	99	24.1 (21.6)	19.8 (18.6)	0.016*
Amount of social contacts	101	36.3 (27.8)	36.4 (28.2)	0.808
Sense of community	102	21.8 (17.1)	21.3 (16.6)	0.881
Self-efficacy	97	33.4 (3.9)	33.5 (3.0)	0.939
TCC	101	7.9 (2.0)	5.3 (2.0)	0.006*

*SD* standard deviation, *TCC* technology competence conviction

*Statistical significance

**Table 5:** Stressors and resources for mobile workers and non-mobile workers, T0 and T1; Mann–Whitney-U-Test (two independent samples)

	T0			T1		
	Mobile work (*N* = 59)	No mobile work (*N* = 43)	*p*	Mobile work (*N* = 90)	No mobile work (*N* = 12)	*p*
	Mean (SD)	Mean (SD)		Mean (SD)	Mean (SD)	
*Stressors*						
Quantitative demands	49.9 (19.7)	40.9 (20.8)	0.027	42.4 (17.6)	36.1 (21.7)	0.242
Emotional demands	57.2 (23.0)	41.9 (29.9)	0.021	48.5 (25.7)	47.9 (31.0)	0.987
Work–privacy conflicts	26.8 (21.7)	18.9 (21.5)	0.047	17.4 (18.9)	9.4 (17.0)	0.123
Trust and justice	47.7 (18.3)	42.2 (17.9)	0.116	39.6 (18.0)	38.0 (17.4)	0.581
Dissolution	20.0 (18.9)	11.3 (17.2)	0.008	17.6 (18.7)	19.8 (25.8)	0.899
Role conflicts	37.9 (21.1)	33.7 (20.7)	0.307	33.1 (19.8)	31.3 (18.5)	0.882
*Resources*						
Influence at work	43.5 (19.6)	61.7 (21.7)	< 0.001	46.4 (19.8)	51.4 (16.2)	0.368
Meaning of work	38.8 (28.6)	57.6 (45.8)	0.040	22.8 (16.3)	25.0 (17.7)	0.492
Predictability of work	44.3 (22.1)	50.3 (19.8)	0.118	37.8 (18.7)	40.6 (10.8)	0.427
Role clarity	31.6 (20.2)	26.2 (22.7)	0.063	24.7 (18.2)	18.1 (16.6)	0.108
Quality of leadership	50.3 (25.4)	54. 4 (29.6)	0.566	42.1 (20.5)	37.5 (20.5)	0.590
Support at work	23.8 (20.0)	24.6 (24.0)	0.846	20.3 (18.7)	16.1 (18.7)	0.368
Amount of social contacts	38.1 (27.6)	33.7 (28.3)	0.405	37.4 (28.5)	29.2 (25.7)	0.400
Sense of community	20.3 (13.9)	23.8 (20.7)	0.667	21.8 (16.6)	17.7 (17.2)	0.494
Self-efficacy	33.9 (3.3)	32.6 (4.5)	0.733	33.5 (3.1)	33.3 (2.9)	0.945
technology competence conviction	4.7 (1.5)	5.2 (2.4)	0.105	5.2 (1.8)	6.5 (3.2)	0.240

*SD* standard deviation, *p* *p* value

Mobile work and workplace stressors/resources: in comparison between mobile workers and non-mobile workers (Tab. 5), mobile workers generally reported more stressors (quantitative demands, emotional demands, work–privacy conflicts, and dissolution) and more resources (exceptions: role clarity, quality of leadership, amount of social contacts, differences not statistically significant) compared to office workers. But this effect may also be attributed to the working areas of office workers and has to be regarded in unison with other factors of the multivariate model. At T1, we found no statistically significant differences in workplace factors between stressors, and workplace and personal resources (Table 5).

**Fig. 2:**
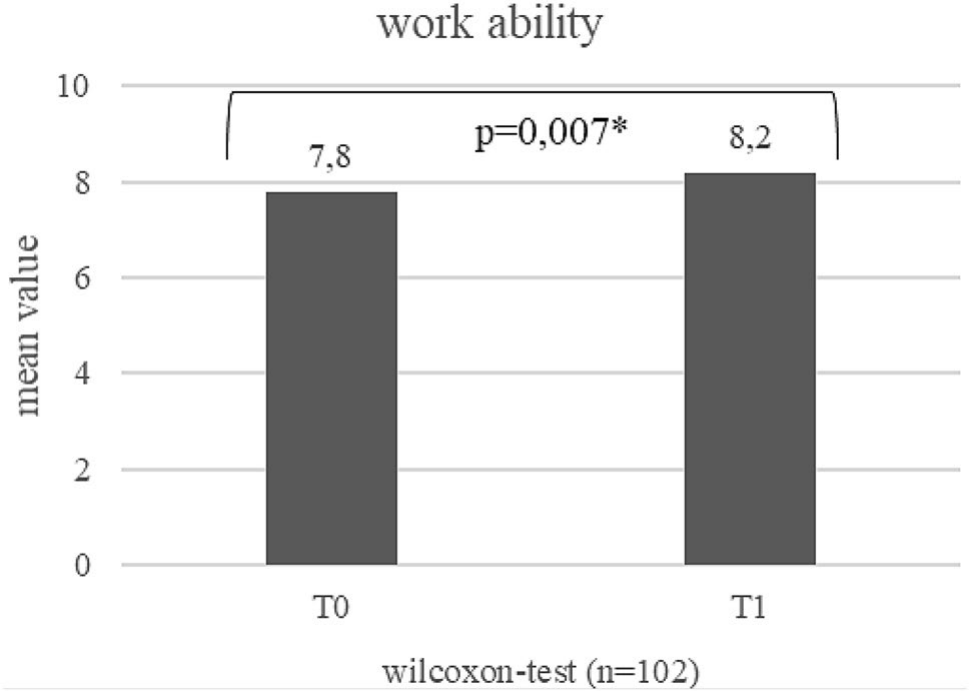
Development of work ability (mean values) during the course of the study (T0–T1)¸ Wilcoxon-test

**Fig. 3:**
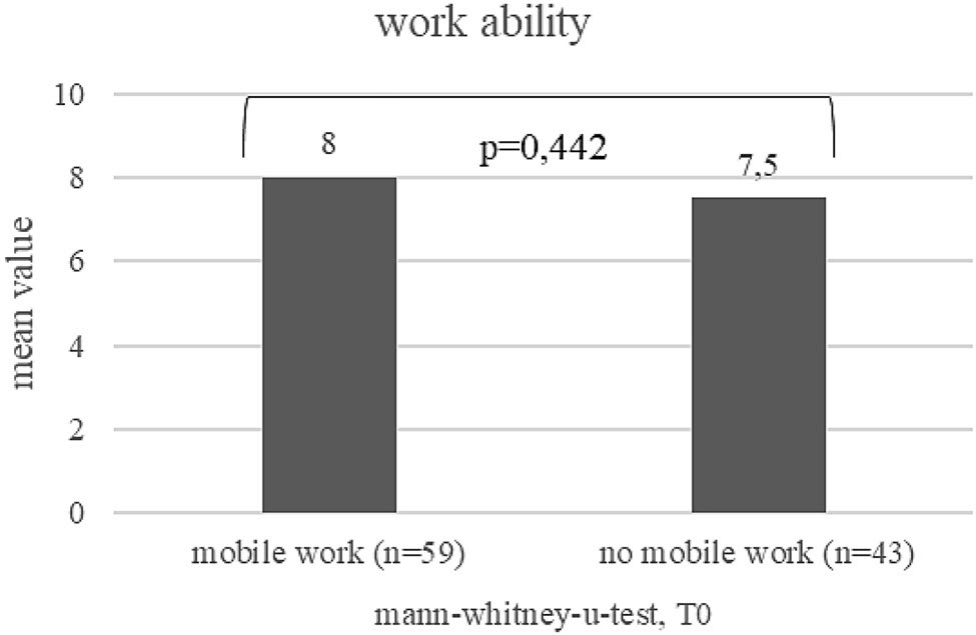
Comparison of work ability (mean values) between mobile workers and office workers at T0; Mann–Whitney-U-test

**Fig. 4:**
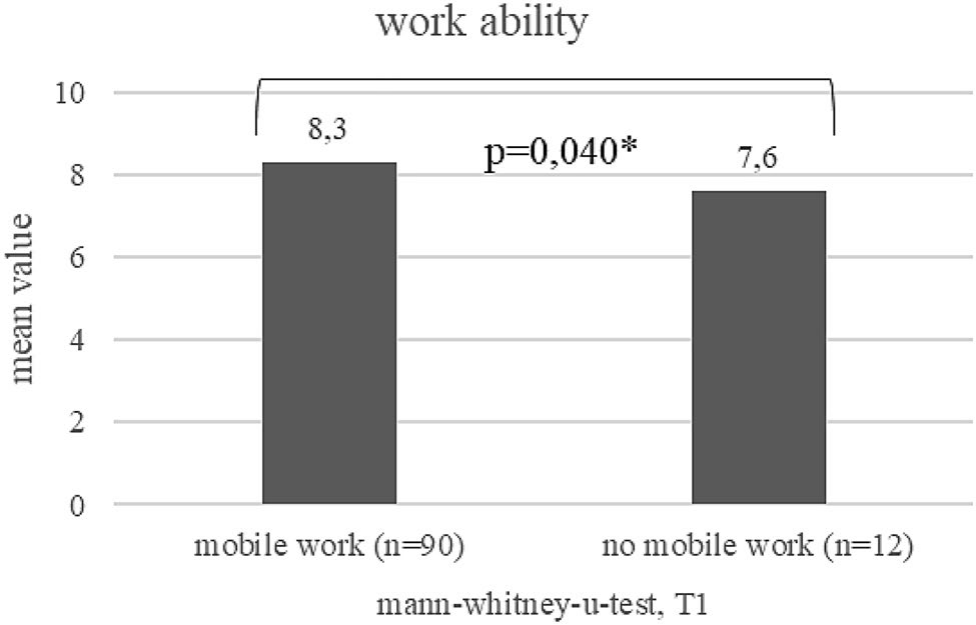
Comparison of work ability (mean values) between mobile workers and office workers at T1; Mann–Whitney-U-test

**Fig. 5:**
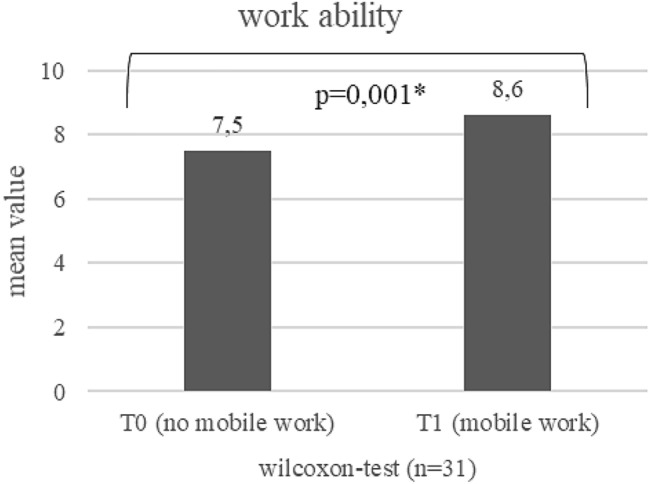
Development of work ability (mean values) of persons not working mobile at T0 and working mobile at T1, Wilcoxon-test

Work ability: in the course of the study, work ability increased between T0 and T1 from on average 7.8–8.2 (*p* = 0.007) (see Fig. 2). Work ability of mobile workers seems to be better than that of non-mobile workers at T0 and T1 (see Figs. 3 and 4). At T1, this difference is statistically significant (*p* = 0.040). The increase was pronounced in persons who did not work mobile at T0, but worked mobile at T1 (*p* = 0.001; Fig. 5).

Workplace stressors/resources, personal resources, and work ability: Table 6 shows the correlations between self-reported work ability and work-related stressors/resources and personal resources, separately for T0 and T1. In detail, there are significant correlations between work ability and quantitative demands and work–privacy conflicts at T0. The fewer the conflicts and the lower the quantitative demands, the better the work ability. At T1, of all examined stressors, only work–privacy conflicts continue to show a significant correlation to work ability.

**Table 6:** Associations between self-reported work ability and stressors and resources, T0 and T1

	T0		T1	
	*r*	*p*	*r*	*p*
*Stressors*				
Quantitative demands	− 0.276	0.005*	− 0.046	0.645
Emotional demands	− 0.129	0.197	− 0.162	0.103
Work–privacy conflicts	− 0.384	< 0.001*	− 0.356	< 0.001*
Trust and justice	− 0.197	0.052	− 0.117	0.245
Dissolution	− 0.053	0.598	− 0.125	0.213
Role conflicts	− 0.183	0.067	− 0.162	0.105
*Resources*				
Influence at work	− 0.344	< 0.001*	− 0.114	0.259
Meaning of work	− 0.046	0.647	− 0.026	0.796
Predictability of work	− 0.149	0.134	− 0.175	0.081
Role clarity	− 0.048	0.631	− 0.057	0.573
Quality of leadership	− 0.285	0.004*	− 0.169	0.091
Support at work	− 0.16	0.112	− 0.276	0.005*
Amount of social contacts	− 0.304	0.002*	− 0.158	0.114
Sense of community	− 0.303	0.002*	− 0.287	0.003*
Self-efficacy	0.279	0.005*	0.185	0.066
TCC	− 0.16	0.107	− 0.354	< 0.001*

*N* = 102

*r* correlation coefficient, *p* *p* value, *TCC* technology competence conviction

*Significant results

Furthermore, there are some resources that correlate significantly with work ability. Influence at work (*p* < 0.001) and work-related social aspects (e.g., quality of leadership, amount of social contacts) in particular play a major role at T0. At T1, sense of community (− 0.287, *p* = 0.003) and support at work (− 0.276, *p* = 0.005) show a significant correlation with the ability to work. With regard to personal resources, higher technology competence conviction was significantly correlated with good work ability at T1 (− 0.354, *p* < 0.001).

Final models for T0 and T1—mobile work and work ability: to gain more insight into the impact of mobile work on work ability, two binary logistic regression models (stepwise backwards) were used (Table 7). They revealed three significant predictors for good work ability at T0 and T1, respectively.

**Table 7:** Binary logistic regression analyses for self-reported work ability, T0 and T1 (backwards selection, likelihood ratio)

	T0^a^		T1^b^	
	OR	95% CI	OR	95% CI
Mobile work			6.04	1.26–28.92*
Work–privacy conflicts	3.38	1.17–9.75*	3.85	1.29–11.53*
Influence at work	5.43	1.70–17.30*		
Quality of leadership	3.91	1.07–14.31*		
Support at work			2.59	0.90–7.47
Amount of social contacts	2.76	0.93–8.23		
Sense of community	0.28	0.07–1.03		
TCC			3.05	1.04–8.94*
*Model quality criteria (final step)*	*p*	*R*^2^	*p*	*R*^2^
	0.002*	0.349	< 0.001*	0.372

*N* = 102

*OR* Odds ratio, *95% CI* 95% confidence interval, *p* p value, *R*^*2*^ model quality, *TCC* technology competence conviction

^a^Model variables T0: mobile work, age, sex, working area, leadership, child in the household, quantitative demands, work–privacy conflicts, influence at work, quality of leadership, amount of social contacts, sense of community, self-efficacy

^b^Model variables T1: mobile work, age, sex, working area, leadership, child in the household, work–privacy conflicts, support at work, sense of community, TCC

*Significant results (*p* < 0.05)

Before the COVID-19 pandemic (T0), self-reported work ability is significantly determined by work–privacy conflict (CI 1.17–9.75, OR 3.38), quality of leadership (CI 1.07–14.31, OR: 3.91) and influence at work (CI 1.70–17.30, OR 5.43). Note that mobile work as independent factor does not influence work ability at T0. Nevertheless, mobile work influences the effect of work–privacy conflict and influence at work.

At T1, the odds for good work ability is 6 times higher when participants work mobile (CI 1.26–28.92, OR 6.04). In addition, good work ability is associated with less work–privacy conflicts (CI 1.29–11.53, OR 3.85) and good technology competence conviction (CI 1.04–8.94, OR 3.05). Both models were adjusted for confounders: age, sex, working area, leadership, and child living in the household.

## Discussion

Over the course of our study, a generally positive correlation between mobile work and work ability was reported. Furthermore, a positive development of work ability, especially for mobile workers between T0 and T1 was found, with mobile workers reporting better work ability compared to non-mobile workers at T0 (not statistically significant) and at T1 (statistically significant). The increase in workability between T0 and T1 is especially interesting, as T1 encompassed the time during the first COVID-19 lockdown in Germany, which was often accompanied by a high personal stress perception in other occupational cohorts (Sandoval-Reyes et al. 2021).

At T0, our results—in general—fit in with the results of other studies, which report better productivity and efficiency of employees who experienced an increase in mobile work (Gajendran and Harrison 2007; Kunze 2020; Sandoval-Reyes et al. 2021; Waltersbacher et al. 2019). Similarly, a study by Hill et al. (2003) identified working from home as predictor for work motivation. This might also explain some of our results at T0, as many employees of the examined company had a generally strong wish for mobile work, and probably wanted to make the pilot phase to become a success. Nevertheless, there were also several employees declining mobile work in general, which could explain the statistical non-significant effect at T0.

As other studies have shown (Oakman et al. 2020; Wang et al. 2021), too, at T0, social components played an important role for work ability. The amount of social contacts and the sense of community at T0 correlate with good work ability. Interestingly, though, we found no statistical difference in these workplace resources with regard to mobile work, despite reports which found that mobile work is associated with a decrease in work-related social contacts and an increase in isolation (Bentley et al. 2016). One explanation can be that our cohort has a low turnover rate and employees work together for many years. In these cases, mobile work can be founded on an existing social understanding between employees and supervisors which is probably beneficial for the introduction of remote work. In the long term, measures need to be taken to stabilize this social network when allowing for mobile work. Having said this, please note that only quality of leadership remained as independent, statistically significant “social” variable for work ability in the adjusted regression model at T0.

At T1, the correlation between “amount of social contacts” and work ability was not statistically significant. The reason for this could be that the resource “amount of social contacts” was seen rather as a stressor during times of a pandemic. Meeting many people personally could pose a higher risk of infection. Contrary to T0, social support was significantly correlated with work ability at T1. This can indicate that the importance of workplace-related social support increases in times of a common and work-related crisis. Companies, therefore, should create means for mobile workers to have access to support even when they are not in the office. This result is also supported by the study of Shimura et al. (2021) which indicates that resources like social support have to be taken into account if remote work is to be successfully introduced at workplaces. There was no statistical difference in the perception of work-related social resources between mobile workers and office workers. It has to be taken into account, though, that the number of persons remaining at the office was rather small. Nevertheless, even in the adjusted regression model, there were no significant interactions between social resources and work ability at T1.

An important influencing variable for good work ability are work–privacy conflicts at T0 and T1. People who are able to combine their private and professional lives well are better able to concentrate on their work and consequently have a better ability to work. This factor remained a stable and significant variable in the adjusted regression models, was important for both time points, and seems to address both effects of mobile work (individual/societal motives vs. work-related stressors and resources). There are other studies, though, which report decreased productivity and a diminished work–life balance of mobile workers (Sandoval-Reyes et al. 2021), as mobile work seems to be able to produce ambiguous effects. In this context, it should be mentioned that work–privacy conflicts at T0 are moderated by mobile work, whereas we found no moderating effect at T1, when mobile work remained as independent variable in the adjusted regression model.

As personal resource, technology competence conviction at T1 has a significant influence on work ability. Due to COVID-19, the independent use of digital communication media and work tools has become more important, which can explain that technology competence conviction can predict good work ability at T1.

In our project, several explanations seem plausible for the positive development of work ability in the context of mobile work. First, it is possible that an existing positive effect of mobile work on work ability was not yet fully measurable at T0, as the test persons had only recently (a few weeks earlier) started to work mobile. At the beginning of this change process, there may be various difficulties, for example regarding work organization or providing the necessary equipment. These difficulties could have decreased an eventually statistically positive effect at the beginning of the study, while they came into full effect with a longer study duration.

Another explanation could be found in context. Under “normal” circumstances (T0), mobile work seems to affect work ability via altered workplace factors (e.g., quantitative demands, work–privacy conflict, and quality of leadership). But prior studies reported that the effects of altered workplace factors on work ability and health are rather small (Burr et al. 2022; Weber et al. 2021). In the context of a pandemic, mobile work can directly affect work ability as it can pose a means for being able to work. In addition, people working from home may have felt safer and less prone to infection and, therefore, could better concentrate on their daily tasks. These individual/societal motives can be directly addressed by mobile work as an independent factor. Therefore, this effect of mobile work on work ability seems be stronger than the mediated effect via workplace factors. These thoughts may be able to explain some of the ambiguous results found in the context of mobile work. Our results once more hint at work ability being a construct which can be influenced by many factors. They also suggest that these factors might express their influence context-specific. With regard to our explorative research focus at company level, we found that work ability can be associated with mobile work itself or be associated with alterations of workplace factors because of mobile work. These findings need to be reproduced in larger cohorts and under more stable study conditions.

Finally, and in the context of healthy mobile work design, the intensity of mobile working must also be taken into account, when discussing the results. A Spanish study indicates that people, who work only occasionally (less than a few times a month) from home, have the best quality of work, whereas people, who work very much (more than a few times a week) from home, have the worst quality of work and work–life balance. Therefore, it is not just a question of whether people work mobile, but also the extent to which they work from home (Rodríguez-Modroño and López-Igual 2021). Similarly, Shimura et al. (2021) found that full remote work (5 days/week) led to a reduction in productivity due to increased presentism. The inhibition threshold to work sick in the home office is very low, since the workplace is not far away, work can be done more flexibly, e.g., starting work later, and there is no danger of infecting colleagues. This is problematic because remote work is associated with an increase in presenteeism. The greater the intensity of remote work, the higher the probability of presenteeism (Steidelmüller et al. 2020). Non-full remote work, on the other hand, shows positive effects in terms of psychological and physical stressors. The results of our study at T1 can, therefore, also possibly be attributable to the pandemic situation which was associated with about 4 days of mobile work per week.

A critical appraisal of our study reveals the following limitations: It must be taken into account that participation in the study was exclusively voluntary at both survey times. Therefore, it is possible that primarily employees, who are highly motivated to work mobile, took part. Since the desire for mobile work has been strong in the company prior to the study, it is possible that the participants answered too positively so as not to jeopardize the introduction of mobile work. In addition, research on mobile work involves various hurdles. Already in 2002, Bailey and Kurland highlighted the problem that despite various studies, the effects of mobile work are unclear. One potential challenge of this research area is that there is often a bias that many study participants wish to work from home.

Furthermore, the meaningfulness of the study results is limited by the relatively small sample. Not all participants took part in both parts of the survey. Thus, the matched cases for T0 and T1 amount to 102 persons. Another possible bias is that the intended ratio between mobile and non-mobile workers changed at T1 according to lockdown restrictions, so that statistical group comparisons at T1 are only of limited value. Nevertheless, by adjusting the final models to these biased variables, we tried to equalize results—though only statistically.

The onset of the COVID-19 pandemic can also have biased other results. Many employees were unpreparedly rushed into the mobile work. Possible negative aspects can, therefore, also be explained by the fact that the starting conditions were not optimal and there was no time for the necessary preparations, such as appropriate technical equipment for mobile word. On the other hand, negative aspects may not have been reported or may not have been perceived as serious, since the employees were grateful that they were able to continue working at all during this time and did not have to worry about losing their jobs.

The observation period of 12 months is relatively short for assessing a change process, so another wave of surveys would be useful to consolidate the results. Some changes would probably only be measurable later, or a certain volatility of results could become obvious—with changing individual and work-related factors. In addition, given that the second wave of the survey took place at the beginning of the COVID-19 pandemic, it would be interesting to find out how the results develop in a further survey after living with the pandemic has become the new normal or working conditions really returned to normal. This would make it possible to differentiate even more clearly which effects can be attributed to mobile work and which to the pandemic situation. A more homogeneous group constellation in terms of sociodemographic data in the comparison groups would also further sharpen the statements on the effects of mobile work on work ability, since confounding variables would be minimized.

On the other hand, the study also comes along with several strengths: The effect of mobile work on work ability is an important issue, which is seldom examined in a longitudinal design. Our cohort represents a typical workforce in German offices—under the impact of demographic change and few new colleagues (because of a lack of qualified personnel in more remote areas). Offering and evaluating mobile work was, therefore, a genuine issue in this company for making working conditions more attractive while retaining productivity and work ability. Finally, the fact that the second survey inadvertently fell at the onset of the COVID-19 pandemic can also be interpreted as a strength of this study. This provided a unique opportunity to directly compare data from before the pandemic with data during the pandemic. In addition, the pandemic brought a significant increase in attention to the topic of “healthy” mobile work.

In summary, it can be said that in our cohort and under our study conditions, mobile work was positively associated with work ability. While mobile work affected work ability, e.g., via work–privacy conflict, a factor on the interface between personal/societal motivation and work-related stressors and resources at T0, it turned out to remain as an independent predictive factor for work ability in the regression model at T1. The altered relevance of mobile work in the model is interesting for further consideration. While technical competence was no important issue at T0, it remained as statistically significant factor in the final regression model at T1. This hints at a need for schooling and training in VUCA times or rather in preparation for VUCA situations. In times of the COVID-19 pandemic, the aspects of work–privacy conflicts and technology competence conviction are gaining importance. In the context of a crisis and increased work–privacy stresses due to COVID-19 pandemic (closure of child care and general care institutions), mobile work seems to be a valuable option for companies to maintain employees' ability to work. Nevertheless, the workplace design for mobile work needs to be adjusted to the specific company, personal and societal motives, work-related stressors and resources, as well as personal competencies. Creating the necessary tailored measures seem to be a major challenge for further developments.

## Declarations

### Availability of data and material

The datasets generated and/or analyzed during the current study are available from the corresponding author on reasonable request.
